# Lansoprazole use and tuberculosis incidence in the United Kingdom Clinical Practice Research Datalink: A population based cohort

**DOI:** 10.1371/journal.pmed.1002457

**Published:** 2017-11-21

**Authors:** Tom A. Yates, Laurie A. Tomlinson, Krishnan Bhaskaran, Sinead Langan, Sara Thomas, Liam Smeeth, Ian J. Douglas

**Affiliations:** 1 Institute for Global Health, University College London, Institute of Child Health, London, United Kingdom; 2 London School of Hygiene & Tropical Medicine, London, United Kingdom; University of California San Francisco, UNITED STATES

## Abstract

**Background:**

Recent in vitro and animal studies have found the proton pump inhibitor (PPI) lansoprazole to be highly active against *Mycobacterium tuberculosis*. Omeprazole and pantoprazole have no activity. There is no evidence that, in clinical practice, lansoprazole can treat or prevent incident tuberculosis (TB) disease.

**Methods and findings:**

We studied a cohort of new users of lansoprazole, omeprazole, or pantoprazole from the United Kingdom Clinical Practice Research Datalink to determine whether lansoprazole users have a lower incidence of TB disease than omeprazole or pantoprazole users. Negative control outcomes of myocardial infarction (MI) and herpes zoster were also studied. Multivariable Cox proportional hazards regression was used to adjust for potential confounding by a wide range of factors. We identified 527,364 lansoprazole initiators and 923,500 omeprazole or pantoprazole initiators. Lansoprazole users had a lower rate of TB disease (*n* = 86; 10.0 cases per 100,000 person years; 95% confidence interval 8.1–12.4) than omeprazole or pantoprazole users (*n* = 193; 15.3 cases per 100,000 person years; 95% confidence interval 13.3–17.7), with an adjusted hazard ratio (HR) of 0.68 (0.52–0.89). No association was found with MI (adjusted HR 1.04; 95% confidence interval 1.00–1.08) or herpes zoster (adjusted HR 1.03; 95% confidence interval 1.00–1.06). Limitations of this study are that we could not determine whether TB disease was due to reactivation of latent infection or a result of recent transmission, nor could we determine whether lansoprazole would have a beneficial effect if given to people presenting with TB disease.

**Conclusions:**

In this study, use of the commonly prescribed and cheaply available PPI lansoprazole was associated with reduced incidence of TB disease. Given the serious problem of drug resistance and the adverse side effect profiles of many TB drugs, further investigation of lansoprazole as a potential antituberculosis agent is warranted.

## Abstract 2

Ian Douglas and colleagues report lower incidence of TB disease in users of the proton pump inhibitor lansoprazole using data from a large UK cohort study.

## Author summary

**Why was this study done?:**

- A recent report describes preclinical laboratory findings showing lansoprazole has strong activity against *M*. *tuberculosis*, including drug-resistant strains.
- Other proton pump inhibitors, omeprazole and pantoprazole had no such activity.
- No clinical investigations of this possible protective association with lansoprazole have yet been reported.

**What did the researchers do and find?:**

- We studied a cohort of new users of lansoprazole, omeprazole, or pantoprazole from the United Kingdom Clinical Practice Research Datalink to determine whether lansoprazole users have a lower incidence of tuberculosis disease than omeprazole or pantoprazole users.
- Comparing 527,364 lansoprazole initiators with 923,500 omeprazole or pantoprazole initiators, lansoprazole users had a lower rate of TB disease with an adjusted HR of 0.68 (0.52–0.89).
- No association was found with negative control outcomes; myocardial infarction (adjusted HR 1.04; 95% confidence interval 1.00–1.08) or herpes zoster (adjusted HR 1.03; 95% confidence interval 1.00–1.06).

**What do these findings mean?:**

- In vitro, animal, and, now, clinical epidemiological data, all suggest that lansoprazole has activity against *M*. *tuberculosis*.
- Pharmacodynamic and early phase clinical trials are warranted to assess whether lansoprazole, or its metabolites, might have a role in the prevention or treatment of *M*. *tuberculosis* infection or tuberculosis disease.

## Introduction

In 2015, there were an estimated 10.4 million incident cases of tuberculosis (TB) globally resulting in approximately 1.4 million deaths [1]. There is little commercial or public investment in TB research and there are only six novel compounds currently in the TB drug development pipeline [2]. In 2015, there were an estimated 480,000 cases of multidrug-resistant TB [1]. Treatment regimens for drug-resistant TB (DRTB) are long and unpleasant, with serious side effects and poor outcomes [1,3,4].

Using a high throughput fibroblast survival assay [5], Rybniker and colleagues found the proton pump inhibitor (PPI) lansoprazole had activity against *M*. *tuberculosis* (MTB), including drug resistant isolates [6]. This activity was confirmed in murine models and was found to result from inhibition of the mycobacterial cytochrome bc_1_ complex, disrupting the respiratory chain [6]. Omeprazole and pantoprazole, other PPIs, had no activity against MTB. This may be attributed to lansoprazole being the only PPI with no substitutions on the benzimidazole ring. Such substitutions are not known to influence treatment efficacy for any existing PPI indication.

Developed for the treatment and prophylaxis of diseases exacerbated by gastric acid production, PPIs are among the most widely used drugs globally. Their side effect profile is favourable compared with drugs used to treat TB [7]. We used the United Kingdom Clinical Practice Research Datalink (CPRD) to compare the incidence of TB disease among individuals taking lansoprazole with that among individuals taking omeprazole or pantoprazole.

## Methods

### Ethics

The study was approved by the London School of Hygiene & Tropical Medicine ethics committee (ref: 11880) and the Independent Scientific Advisory Committee (ISAC) of the Medicines and Healthcare Products Regulatory Agency. The final study protocol was made available to journal reviewers and is attached as supplementary material (S1 Protocol).

### Data sources

The CPRD contains anonymised data from UK general practitioners and includes approximately 8% of the UK population [8]. Information includes comprehensive recording of consultations, diagnoses, prescribed medicines, and basic demographics. Practices and patients are broadly representative of the UK population [8], and data quality is subject to rigorous audit. The data have been used to conduct over 900 published studies, and data validity has been shown to be high for a variety of diagnoses; rates of recorded TB are similar to notification data from Public Health England, suggesting good case ascertainment [9]. Over 50% of CPRD patients have their general practice records linked to Hospital Episode Statistics (HES). HES include all inpatient National Health Service (NHS) hospitalisations (coded using International Classification of Diseases [ICD]-10) [10]. The January 2015 version of CPRD was used (data available 9th Sep 1987–5th Jan 2015).

### Study population and exposure

All patients aged 16 years or over with at least 12 months research-quality follow up in the CPRD were eligible. From this group, we identified a) all new lansoprazole users and b) all new omeprazole or pantoprazole users. In both cohorts, patients had to have at least 12 months prior registration with no previous record of receiving any PPI. No limit on minimum duration of PPI exposure was made. We used prescribing records to determine intended treatment duration for each prescription, and imputed the population level median if this information was missing. In the UK, long term courses of medication are issued in small batches, each batch being covered by an individual prescription. A typical prescription provides enough medication to last 1 month, and so a 12-month course of treatment would usually involve 12 prescriptions. Although PPIs are available without prescription in the UK, this is unlikely to influence our estimates of exposure as it is doubtful many patients would be prescribed a PPI and simultaneously obtain a different one without prescription. Patients were excluded if they: had <12 months prior follow up; prior use of a different PPI; were aged <16 years at start of PPI treatment; or prior diagnosis of TB.

### Outcome

All clinical records indicating TB disease were extracted using Read Codes listed in Supplementary material (S1 Text). The earliest record was taken as the date of diagnosis. It is well recognised that there can be a considerable delay between infection with *M*. *tuberculosis* and diagnosis of TB disease. This means the aetiologically relevant exposure is likely to be earlier than the recorded diagnosis. In a Dutch study, amongst people diagnosed with TB, the median period between infection and diagnosis was 1.26 years, albeit with substantial variability [11]. For these reasons, all TB onset dates were moved earlier by 12 months in the primary analysis. Follow-up time for TB noncases was also censored 12 months earlier, as the final period of follow up would otherwise become ‘immortal’ time during which an outcome could not occur. Patients with <12 months follow up therefore contributed no follow up to the primary analysis. Supplementary material (S1 Fig) depicts how typical patient timelines were affected by this offset.

Approximately three-quarters of TB cases in England are among foreign-born individuals [12]. Given the higher force of infection in most countries of origin, it is likely that much of this TB results from reactivation of latent infection acquired abroad. The interval between arrival in England and TB diagnosis varies considerably by country of birth [12]. Among well-established migrant communities, TB cases may be a result of infection many years prior. The biology of latent MTB infection is poorly understood [13] and, to our knowledge, there have been no attempts to quantify the interval between ‘reactivation of latent infection and onset of symptoms or TB diagnosis. Biologically, the doubling time of MTB might be expected to be similar in both instances, and the mycobacterial burden required for symptoms to become apparent might also be expected to be similar. This logic would suggest that the same offset should be applied. However, given the considerable uncertainty surrounding this assumption, sensitivity analyses were undertaken (see below). Any misclassification of exposure status might be expected to bias our effect estimate towards the null.

PPI users are likely to be less healthy than nonusers [14]. The choice of omeprazole and pantoprazole users as the comparator group should mean we are comparing groups of people with similar health at baseline. These drugs are considered by most clinicians to be essentially interchangeable. However, if perceived health also influences the choice of individual PPI, this could be difficult to detect and account for. To guard against the possibility that any lansoprazole effect is driven by an unmeasured imbalance in baseline health, as a posthoc check we analysed the cohort for two additional ‘control’ outcomes; myocardial infarction (MI; associated with poor health) and herpes zoster (associated with impaired immunity). There is no reason to suspect either outcome is influenced by PPI choice. Each outcome was defined as the first record of this outcome in the CPRD clinical or referral record.

### Covariates

Covariates explored as potential confounders included age, sex, calendar year, smoking behavior, body mass index (BMI), alcohol use, ethnicity, drug abuse, any prior use of inhaled/oral corticosteroids, travel vaccines and antimalarial prescriptions (proxies for travel to TB endemic regions), diabetes, poorly controlled diabetes (one or more measure of HbA1c > 9% in the previous year), rheumatoid arthritis (RA), inflammatory bowel disease (IBD), chronic obstructive pulmonary disease, asthma, chronic kidney disease (CKD), depression, leukaemia, lymphoma, myeloma, and recorded HIV infection. We also adjusted for two variables measured at the practice level: the proportion of new PPI users given lansoprazole each year and the index of multiple deprivation (IMD) score.

Of note, ciclosporin and tacrolimus, both immunosuppressive drugs, are thought to interact with omeprazole but not other PPIs. It is possible that users of these drugs (who are probably at higher risk of TB) will be given PPIs other than omeprazole. For this reason, patients with a history of use of either drug were excluded from the study population.

### Statistical analysis

Each participant’s follow up time began at the first prescription for lansoprazole or omeprazole/pantoprazole. All subsequent time was classified as follows:

- *Lansoprazole–*Time covered by lansoprazole prescriptions
- *Omeprazole/pantoprazole–*Time covered by omeprazole/pantoprazole prescriptions. This was the baseline against which lansoprazole exposure was compared.
- *Unexposed*–All time between lansoprazole/omeprazole/pantoprazole exposure periods (treatment breaks), or between the end of PPI exposure and the end of follow up. This time is excluded from the main comparison of interest as time off a PPI may represent periods of better health, and a corresponding change in the risk of TB disease

PPI exposed time included a 60-day period after estimated treatment end date, allowing for stock piling and nonadherence. People starting treatment with omeprazole/pantoprazole but later receiving lansoprazole transitioned to the lansoprazole group at that time and vice versa. End of follow up was the earliest of first recorded TB disease, death, transfer to a different general practice, or last data collection date.

Cox regression was used to estimate hazard ratios (HRs) and 95% confidence intervals, comparing all lansoprazole-exposed time against all omeprazole- or pantoprazole-exposed time. A crude model was constructed with just the main exposure variable, followed by a model adjusting for all potential confounders with complete data. We then investigated potential confounding by variables with missing data (smoking, BMI, alcohol, ethnicity, and CKD). To do this, we constructed models excluding people with missing data on each variable. These variables were only retained in the final model if their inclusion altered the HR by >5%, thereby ensuring the maximum possible sample size was achieved. We ensured that the proportional hazards assumption was met by examining Schoenfeld residuals. An interaction term was fitted to look for effect modification between lansoprazole exposure and age. TB incidence was also measured after PPI treatment had been discontinued in an analysis comparing past lansoprazole users with past omeprazole or pantoprazole users. For the negative control outcome of MI, additional adjustments were made for the following risk factors if they had been recorded at any point prior to starting the PPI: coronary heart disease, cerebrovascular disease, peripheral vascular disease, other atheroma, hypertension, heart failure, and statin use.

The burden of TB disease may vary from practice to practice due to differences in patient populations (e.g., urban versus rural). To see whether this variation was related to the chance of being prescribed lansoprazole, we calculated practice-level TB prevalence (ever recorded diagnosis) and practice-level proportion of lansoprazole, omeprazole, and pantoprazole prescribing accounted for by lansoprazole over the study period. Linear regression was conducted to describe any association between them.

At the request of referees, we explored indications for PPI prescribing. Indication is not recorded or linked with the prescribing record by GPs, so we instead examined diagnoses recorded on the day the PPI was first prescribed.

### Sensitivity analyses

The following sensitivity analyses were undertaken. 1) To address possible delays in diagnosis or in recording the TB diagnosis in the primary care record and the known variability in the incubation period, TB onset was redefined as A) date recorded in CPRD, B) 2 years earlier than recorded, and C) 5 years earlier than recorded. Longer incubation periods than those described in the Dutch study might be expected in first generation migrants infected in their country of origin [11]. 2) CPRD-linked HES inpatient data were searched for ICD10 codes indicating TB (A15–A19). An analysis restricted to CPRD HES-linked practices and time periods was conducted, taking the earliest of CPRD- or HES-identified TB disease. 3) The time following the end of estimated treatment duration at which we assumed therapy ceased was extended from 60 to 90 days. 4) Follow-up was censored after cessation or switch of PPI therapy (cessation defined as longer than 60 days not covered by a prescription). 5) As ethnicity is not recorded for all patients, and is unlikely to be perfectly captured for those with a record, we conducted an analysis restricted to patients recorded as having any white ethnicity. 6) Very short courses of lansoprazole therapy may not have an impact on TB incidence; we therefore conducted an analysis excluding people who received less than a 28-day supply of PPI over the study period.

## Results

### Background detail

**Fig 1:**
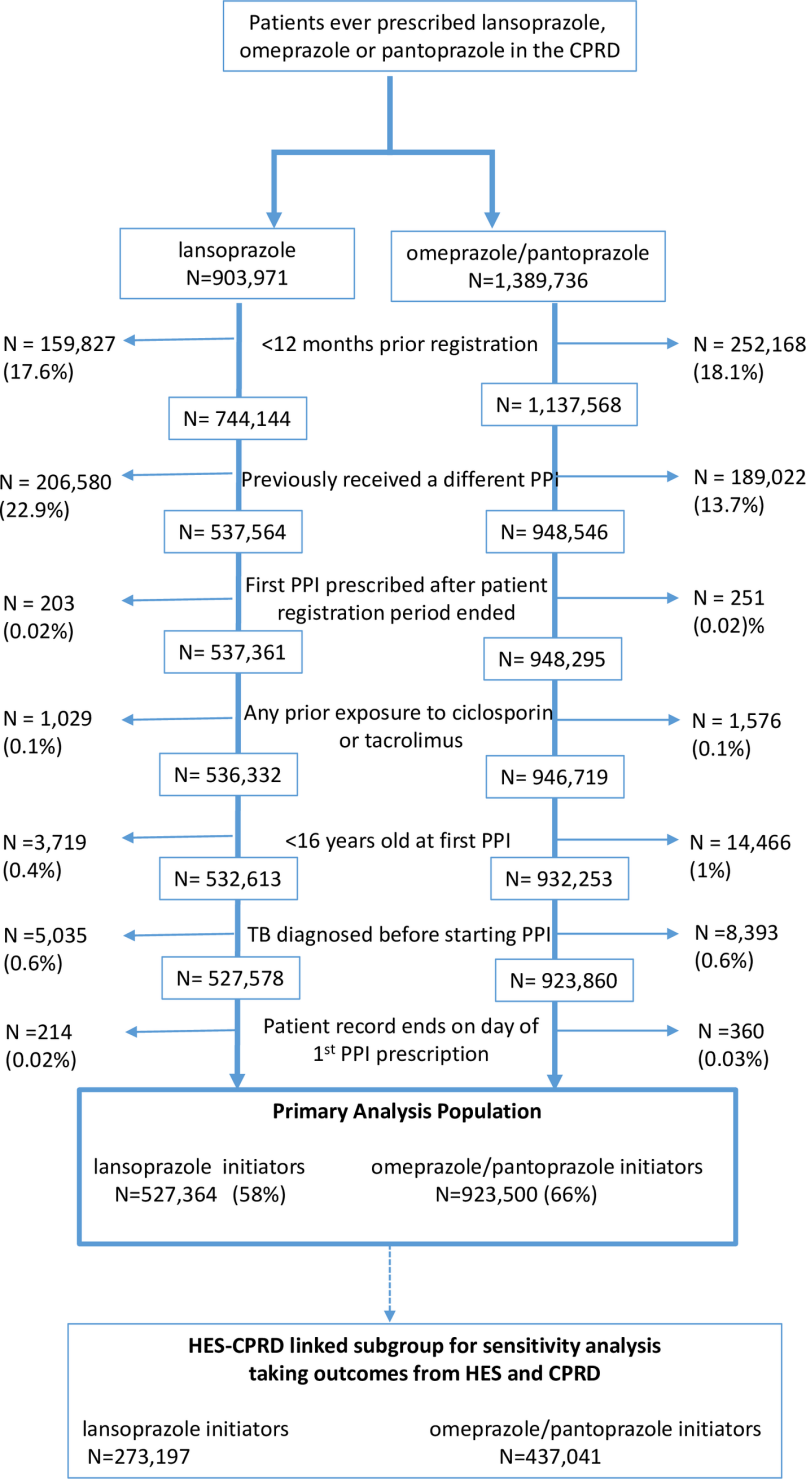
Flow diagram of patient exclusions and eligibility. CPRD, Clinical Practice Research Datalink; HES, Hospital Episode Statistics; PPI, proton pump inhibitor.

There were 527,364 new users of lansoprazole and 923,500 new users of omeprazole or pantoprazole, after exclusions were applied (see Fig 1). The intended treatment duration was missing for 1% of individual PPI prescriptions, and the population median of 28 days per prescription was imputed for these records. Table 1 shows the background characteristics of the patients using data recorded on or before the first PPI prescription. Mean total exposure to PPI was 408 days for lansoprazole users and 386 days for omeprazole or pantoprazole users. Of note, the lansoprazole group had more current smokers (23% versus 16%) and lansoprazole was used less frequently than omeprazole and pantoprazole in more recent years (2006 onwards). Otherwise, the groups were largely similar. The 10 most frequently recorded clinical signs, symptoms, and diagnoses on the day a PPI was first prescribed were all synonyms for dyspepsia, gastroesophageal reflux disease, or abdominal pain. This did not differ between lansoprazole users and omeprazole or pantoprazole users. The mean time between first PPI prescription and first record of TB diagnosis was 1.2 years for lansoprazole users (standard deviation = 2.0) and 1.1 years for omeprazole/pantoprazole users (standard deviation = 1.8).

**Table 1:** Characteristics of PPI users at initiation of either lansoprazole or omeprazole/pantoprazole therapy.

	Lansoprazole (*n* = 527,364)	Omeprazole or pantoprazole (*n* = 923,500)
**Mean Age, yrs (SD)**	56 (18)	54 (18)
**Female, *n*(%)**	288,442 (54.7)	512,242 (55.5)
**Mean Follow Up Post-PPI Initiation, yrs (SD)**	1.0 (1.9)	0.9 (1.6)
**Total Duration of Prescribed PPI *n*(%)**		
Up to 7 days	1,537 (0.3)	3,290 (0.4)
8–14 days	5,038 (1.0)	9,349 (1.0)
15–28 days	23,795 (4.5)	36,206 (3.9)
29–365 days	373,462 (70.8)	655,368 (71.0)
>1 year	123,532 (23.4)	219,017 (23.7)
**Smoking, *n*(%)**		
Non	221,516 (42.0)	398,029 (43.1)
Current	122,672 (23.3)	147,514 (16.0)
Ex	175,320 (33.2)	366,148 (39.6)
Missing	7,856 (1.5)	11,809 (1.3)
**Alcohol Consumption, *n*(%)**		
Non-drinker	71,227 (13.5)	120,410 (13.0)
Ex-drinker	22,152 (4.2)	42,232 (4.6)
Current drinker, unknown quantity	2,519 (0.5)	3,918 (0.4)
<2 units/day	92,659 (17.6)	160,793 (17.4)
3–6 units/day	224,804 (42.6)	384,463 (41.6)
>6 units/day	53,996 (10.2)	97,517 (10.6)
Missing	60,007 (11.4)	114,167 (12.4)
**Calendar Year at PPI Initiation, *n*(%)**		
1989–1990	0 (0.0)	423 (0.1)
1991–1995	2,378 (0.5)	40,127 (7.6)
1996–2000	54,910 (10.4)	62,767 (11.9)
2001–2005	177,443 (33.6)	146,723 (27.8)
2006–2010	181,673 (34.4)	369,359 (70.0)
2011–2015	110,960 (21.0)	304,101 (57.7)
**Ethnicity, *n*(%)**		
White	309,358 (58.7)	541,241 (58.6)
South Asian	9,019 (1.7)	18,581 (2.0)
Black	5,262 (1.0)	9,123 (1.0)
Other	3,923 (0.7)	7,958 (0.9)
Mixed	1,344 (0.3)	2,534 (0.3)
Missing	198,458 (37.8)	344,063 (37.3)
**Comorbidities, *n*(%)**		
Asthma	76,448 (14.5)	133,459 (14.5)
Blood cancer*	4,045 (0.8)	5,786 (0.6)
COPD	20,905 (4.0)	34,066 (3.7)
Depression	115,268 (21.9)	204,669 (22.2)
Diabetes	45,062 (8.5)	76,907 (8.3)
Drug abuse**	6,348 (1.2)	12,917 (1.4)
HIV	225 (0.0)	404 (0.0)
Inflammatory Bowel Disease	5,739 (1.1)	10,466 (1.1)
Rheumatoid arthritis	8,515 (1.6)	12,482 (1.4)
Chronic kidney disease (stage 3 or higher)	34,095 (6.5)	51,748 (5.6)
**Prior Medication (Any Time), *n*(%)**		
Antimalarials	12,585 (2.4)	25,424 (2.8)
Inhaled corticosteroids	96,242 (18.2)	168,164 (18.2)
Oral corticosteroids	86,714 (16.4)	149,416 (16.2)
Travel_vaccines	3,670 (0.7)	6,546 (0.7)
Isoniazid	34 (0.0)	157 (0.0)

*Lymphoma, leukaemia, or myeloma

**Clinical or referral record of any form of drug or alcohol abuse

**Abbreviations:** COPD, chronic obstructive pulmonary disease; PPI, proton pump inhibitor; yrs, years.

In the primary analysis, with recorded TB dates moved earlier by 12 months, the rate of TB was lower with 10.0 cases per 100,000 person-years (pyrs) (95% confidence interval; 8.1–12.4) in people receiving lansoprazole compared with 15.3 cases per 100,000 pyrs (13.3–17.7) in those receiving omeprazole/pantoprazole (Table 2). The crude HR was 0.65 (0.51–0.84), with a fully adjusted HR of 0.68 (0.52–0.89). Censoring follow-up at the first evidence of a treatment break resulted in a similar effect, with an adjusted HR of 0.59 (0.36–0.97). Considering postexposure periods of time when patients received no PPI, there was no detectable difference in TB incidence between patients who had received lansoprazole and those who had received omeprazole/pantoprazole. The HR was 0.94 (0.73–1.20), using the same outcome definition as in the primary analysis (with the outcome date, again, brought forward by 12 months, Table 2).

**Table 2:** Association between lansoprazole and incident TB disease, compared with omeprazole or pantoprazole.

Outcome Exposure Group	Pyrs at Risk (x100,000)	TB Cases (*n)*	Rate of TB Per 100,000 pyrs	Crude HR (95% CI):	Adjusted* HR (95% CI):
***Primary Analysis*: *TB date = CPRD date − 12 months***					
*All PPI exposure included*					
Omep/pantop exposed	12.6	193	15.3 (13.3–17.7)	Referent	Referent
Lansop exposed	8.6	86	10.0 (8.1–12.4)	0.65 (0.51–0.84)	0.68 (0.52–0.89)
*Censored at 1st break*					
Omep/pantop exposed	5.6	76	13.6 (10.9–17.0)	Referent	Referent
Lansop exposed	3.7	32	8.6 (6.1–12.2)	0.63 (0.42–0.95)	0.59 (0.36–0.97)
*Post-treatment periods only*					
Post omep/pantop	22.7	251	11.0 (9.8–12.5)	Referent	Referent
Post lansop	15.0	143	9.6 (8.1–11.3)	0.87 (0.70–1.06)	0.94 (0.73–1.21)
***Sensitivity Analyses***					
***TB date = CPRD recorded date—****All PPI exposure included*					
Omep/pantop	15.7	322	20.5 (18.4–22.9)	Referent	Referent
Lansop	10.4	157	15.1 (12.9–17.7)	0.74 (0.61–0.89)	0.75 (0.62–0.92)
***TB date = CPRD date − 24 months—****All PPI exposure included*					
Omep/pantop	10.0	139	13.9 (11.7–16.4)	Referent	Referent
Lansop	7.1	72	10.2 (8.9–11.0)	0.73 (0.55–0.98)	0.77 (0.57–1.04)
***TB date = CPRD date − 5 years)****—All PPI exposure included*					
Omep/pantop	4.6	67	14.4 (11.3–18.3)	Referent	Referent
Lansop	3.8	34	9.0 (6.4–12.6)	0.63 (0.41–0.95)	0.72 (0.47–1.09)
***TB Sensitivity Analysis*: *definition (earliest of HES and CPRD record − 12 months; note reduced population size due to linkage eligibility)—****All PPI exposure included*					
Omep/pantop	7.1	101	14.2 (11.7–17.3)	Referent	Referent
Lansop	5.1	61	12.0 (9.3–15.4)	0.84 (0.61–1.15)	0.86 (0.61–1.19)
***TB date = CPRD recorded date − 12 months in white patients only—****All PPI exposure included*					
Omep/pantop	7.8	100	12.8 (10.6–15.6)	Referent	Referent
Lansop	5.3	50	9.4 (7.2–12.5)	0.73 (0.52–1.03)	0.72 (0.51–1.02)
***Excluding people with <28 days total supply of PPI—****All PPI exposure included*					
Omep/pantop	12.3	190	15.3 (13.3–17.7)	Referent	Referent
Lansop	8.4	84	10.0 (8.1–12.4)	0.65 (0.50–0.84)	0.67 (0.51–0.88)
***Excluding people with prior isoniazid exposure****—All PPI exposure included*					
Omep/pantop	12.6	193	15.3 (13.3–17.7)	Referent	Referent
Lansop	8.6	85	10.0 (8.0–12.3)	0.65 (0.50–0.83)	0.67 (0.52–0.88)

*Adjusted for age (continuous), sex, calendar year (categorised as 1989–1993, 1994–2012 in single years, 2013–2015), annual practice lansoprazole prescribing proportion (quartile), asthma, blood cancer, COPD, depression, diabetes, poorly controlled diabetes, drug abuse, HIV, IBD, RA, prior use of antimalarials, practice index of multiple deprivation quintile, inhaled corticosteroids, oral corticosteroids, and travel vaccines

**Abbreviations:** CI, confidence interval; COPD, chronic obstructive pulmonary disease; CPRD, Clinical Practice Research Datalink; HES, Hospital Episode Statistics; HR, hazard ratio; IBD, inflammatory bowel disease; PPI, proton pump inhibitor; pyrs: person-years; TB, tuberculosis

All sensitivity analyses produced results consistent with the primary analysis. For the analysis of HES/CPRD TB outcomes, confidence intervals were substantially wider and crossed unity, reflecting the reduced size of the dataset when restricting to patients with linked data (Table 2). In sensitivity analyses including variables recorded only in a subset of participants (IMD, ethnicity and CKD), inclusion of these variables, in a complete case analysis, had little impact on the HR (S1 Table). An analysis restricted to white patients also found similar results (HR = 0.72; 0.51–1.03). There was no evidence of an interaction between lansoprazole use and age, (*p* = 0.90, S2 Table), and the analysis allowing 90 days without a prescription before assuming therapy had ceased gave very similar results to the primary analysis (S3 Table).

Using linear regression, we found no evidence of any association between practice level TB prevalence and the practice level proportion of PPI prescribing accounted for by lansoprazole (*p* = 0.98; see S2 Fig for scatterplot).

For the negative control outcome of MI, patients receiving lansoprazole had very similar rates to those receiving omeprazole/pantoprazole both during periods of PPI exposure (adjusted HR = 1.04; 1.00–1.08, see Table 3), and post-PPI exposure (adjusted HR 0.98 (0.93–1.03). A similar pattern was seen for herpes zoster, with an on-treatment adjusted HR of 1.03 (1.00–1.06), and a post-treatment adjusted HR of 1.01 (0.98–1.04).

**Table 3:** Association between lansoprazole and control outcomes, compared with omeprazole or pantoprazole.

Outcome Exposure Group	Pyrs at risk (x100,000)	Outcomes (*n*)	Rate of Outcome Per 10,000 pyrs	Crude HR (95% CI)	Adjusted HR* (95% CI):
***Myocardial Infarction***					
*All PPI exposure included*					
Omep/pantop	14.9	7,337	49.4 (48.3–50.5)	Referent	Referent
Lansop	9.7	5,215	53.9 (52.5–55.4)	1.06 (1.03–1.10)	1.04 (1.00–1.08)
*Censored at 1st break*					
Omep/pantop	6.5	3,596	55.7 (53.9–57.6)	Referent	Referent
Lansop	4.1	2,475	60.4 (58.1–62.8)	1.04 (0.99–1.10)	1.07 (1.00–1.14)
*Post-treatment period only*					
Omep/pantop	27.5	6,250	22.8 (22.3–23.3)	Referent	Referent
Lansop	17.5	3,950	22.6 (22.0–23.4)	0.97 (0.93–1.01)	1.00 (0.95–1.05)
***Herpes zoster***					
*All PPI exposure included*					
Omep/pantop	14.4	11,948	82.5 (81.1–84.0)	Referent	Referent
Lansop	9.6	8,331	87.1 (85.2–89.0)	1.04 (1.01–1.07)	1.03 (1.00–1.06)
*Censored at 1st break*					
Omep/pantop	6.4	4,978	77.9 (75.8–80.1)	Referent	Referent
Lansop	4.1	3,480	84.4 (81.6–87.3)	1.06 (1.01–1.10)	1.04 (0.99–1.10)
*Post-treatment period only*					
Omep/pantop	26.3	15,151	57.6 (56.7–58.5)	Referent	Referent
Lansop	16.7	9,974	59.5 (58.3–60.7)	1.02 (0.99–1.04)	1.01 (0.98–1.04)

*Adjusted for age (continuous), sex, calendar year (categorised as 1989–1993, 1994–2012 in single years, 2013–2015), annual practice lansoprazole prescribing proportion (quartile), asthma, blood cancer, COPD, depression, diabetes, drug abuse, HIV, IBD, RA, prior use of antimalarials, practice index of multiple deprivation quintile, inhaled corticosteroids, oral corticosteroids and travel vaccines. Additionally, for MI only: cerebrovascular disease, coronary heart disease, peripheral vascular disease, other atheroma, hypertension, heart failure and statin use.

**Abbreviations:** CI, confidence interval; COPD, chronic obstructive pulmonary disease; HR, hazard ratio; IBD, inflammatory bowel disease; PPI, proton pump inhibitor; pyrs, person-years; RA, rheumatoid arthritis.

## Discussion

In a large, validated, and nationally representative dataset, we have demonstrated a protective association between lansoprazole use and newly diagnosed TB disease with an adjusted HR of 0.68 (0.52–0.89) when compared with omeprazole or pantoprazole use. This association was not seen in past users of these drugs and no association was seen between lansoprazole use and our negative control outcomes of MI and herpes zoster.

We selected a cohort of new adult users of lansoprazole, omeprazole, or pantoprazole, and the only clinical exclusion criteria was for patients with previous exposure to ciclosporin or tacrolimus, in order to avoid a potentially biased sample of lansoprazole patients at increased risk of TB disease. This exclusion affected approximately 0.1% of otherwise eligible patients, with all other exclusions based on age, length of time under observation in the CPRD, or prior history of TB. Our estimate of the TB notification rate in the study population is very similar to the 10.5 per 100,000 pyrs measured by Public Health England in 2015 [12]. People prescribed PPIs are not a random sample of the general population; they tend to be older and have more morbidity, especially gastrointestinal disease [14]. Nonetheless, if the association we report here is due to the pharmacological action of lansoprazole described by Rybniker [6], we can think of no biological reason why this effect would not be seen in the wider population.

We did not anticipate identifying many true confounders as, to explain our results, these would need to be associated with both the choice of PPI and TB disease. Whilst multiple risk factors for TB are known, what matters here is whether they are also associated with the choice of one PPI over another. Clinicians consider PPIs broadly equivalent and would generally not consciously select a specific PPI based on patient characteristics. In the UK, choice of specific drugs within a class may be mandated by guidelines for regional groups of general practitioners or influenced by cost, the efficacy of marketing activities, prescriber preference, or habit. One possible alternate explanation for our results is that regional variation in prescribed PPI was associated with local rates of TB infection. However, we found no association between practice level prevalence of TB infection and likelihood of prescribing lansoprazole. Similarly, whilst both choice of PPI and TB incidence varied over time, adjustment for calendar year did not affect our results. We were unable to adjust for some TB risk factors such as country of birth, homelessness, imprisonment, or prior use of biologic therapies. Nonetheless, we were able to adjust for proxy measures of some of these factors such as ethnicity, history of RA or cancer, and socioeconomic status. Indeed, the striking lack of change in the estimated HR when we adjusted for many potential risk factors suggests that there is little confounding. The protective association we report here is consistent with lansoprazole having clinical activity against MTB in humans.

If causal, the protective effect demonstrated may underestimate that which could be achieved in MTB infection or TB disease. For example, although the patients in our analysis would mostly have been prescribed their PPI once daily, perfect adherence to this regimen is unlikely. Adherence to treatment may be better in individuals with a life-threatening infection, taking treatment for a discrete period of time, and receiving appropriate support.

Little previous work has been done to investigate the association between PPI use and TB. However, Hsu et al. [15] found an association between acid suppressing medication and an increased risk of TB disease, which on the surface appears at odds with our findings for lansoprazole. However, the association declined to null with increasing duration of therapy with either a PPI or a histamine H2 receptor antagonist. This points towards reverse causality as a possible explanation, whereby people with unrecognised early symptoms of TB may be prescribed an acid suppressant.

It is not possible to determine from our results whether the association we demonstrated was against TB resulting from recent infection or from reactivation of latent infection. Most TB in England is thought to result from reactivation [12]. Were some individuals to have had early active disease on starting lansoprazole, use of a single drug might have been insufficient to prevent the emergence of resistance during the course of ‘treatment’. TB disease (though not latent infection) is usually treated with a combination of different drugs.

The absence of a persistent effect of lansoprazole after stopping treatment, in the context of a population within which incident TB is most likely due to reactivation of latent *M*. *tuberculosis* infection, suggests that lansoprazole, at these doses, does not sterilise. This is also consistent with the in vitro data, which suggest lansoprazole metabolites are bacteriostatic [6]. Several drugs used in regimens to treat TB disease are also bacteriostatic [16], e.g., cycloserine and para-amino salicylic acid. We note that isoniazid, a key drug used both to treat latent *M*. *tuberculosis* infection and TB disease, did offer long-term protection in early randomised controlled trials in individuals with latent infection [17]. However, recent mathematical modeling studies suggest that *M*. *tuberculosis* infection probably persists in the majority of individuals after a course of isoniazid. These analyses used data from largely HIV positive individuals enrolled in randomised controlled trials in settings with a high burden of TB disease [18–20]. The biology of latent *M*. *tuberculosis* infection is complex and poorly understood [13]. The balance between bactericidal and bacteriostatic activity for particular drugs can vary depending on the dose of drug given and the metabolic state of the mycobacteria [16]. The precise nature of any effect of lansoprazole cannot be ascertained from our data.

The original in vitro work found lansoprazole to be acting as a pro-drug [6]. The metabolite with activity against MTB, lansoprazole sulfide, is produced via intracellular metabolism. Whilst we could not study the direct effects of lansoprazole sulfide, it is a stable metabolite and importantly has no activity against the gastric H^+^ K^+^ ATPase, the PPI drug target [6]. Therefore, it might be used to treat MTB with fewer off-target effects. Lansoprazole and its metabolites have a number of attractive properties. PPIs have a very favourable side effect profile, as compared with drugs currently used to treat TB [7]. There is no evidence that lansoprazole interacts meaningfully with drugs commonly used to treat TB or HIV [21]. Lansoprazole is off-patent with inexpensive generic versions available. In addition, in vitro studies suggest the drug might have activity against both drug sensitive and drug-resistant strains [6].

We were unable to assess whether TB diagnosis was associated with lansoprazole dose, and it is not known how the currently licensed dose of lansoprazole compares with the optimal dose for activity against MTB. The initial in vitro work by Rybniker et al. tested the pro-drug lansoprazole itself. Strong activity against MTB was detected at a lansoprazole concentration of 10 μM and half maximal activity (IC_50_) was observed at 1.47 μM, or 2.2 μM in a second cell line [5,6]. When given orally at a typical daily dose of 30 mg, C_max_ for lansoprazole in human plasma is 1 mg/ml [22], with little change after dosing for five days. This corresponds to a concentration of 2.71μM [23]—i.e., a concentration in plasma greater than the estimated IC_50_. Further work to determine whether effective concentrations of the sulfide metabolite can safely be achieved in relevant tissues in humans may be needed (e.g., in granulomas or pulmonary cavities). However, very high doses of lansoprazole sulfide (300 mg/kg) have been tolerated by mice [6], suggesting it may be possible to treat humans with higher doses than individuals in CPRD would have been receiving.

In this study, use of lansoprazole was associated with a reduced incidence of TB disease compared with omeprazole or pantoprazole. To our knowledge, these are the first observations to suggest that lansoprazole may have clinical activity against MTB in humans. They are consistent with evidence from both in vitro and animal studies [5,6]. Given the problems of antimicrobial resistance and the adverse side-effects seen with many antituberculous agents, these results are welcome. Our results do not directly address the question of whether lansoprazole or its metabolites would be effective as part of a treatment regimen for MTB infection or TB disease. However, since lansoprazole is safe and well-tolerated, there is a strong case for efficacy studies in humans.

## Supporting information

S1 STROBE Checklist (DOC)

S1 Fig Diagrammatic representation of typical patient timelines in the primary analysis. (DOCX)

S2 Fig Scatterplot of practice-level TB prevalence and practice-level lansoprazole prescribing as a proportion of PPI prescribing. PPI, proton pump inhibitor; TB, tuberculosis.
(DOCX)

S1 Text Read Codes indicating TB disease. TB, tuberculosis.
(DOCX)

S1 Table Association between lansoprazole and incident TB disease, compared with omeprazole or pantoprazole; sensitivity analyses adjusting for variables not recorded for all patients. TB, tuberculosis.
(DOCX)

S2 Table Association between lansoprazole and incident TB disease, compared with omeprazole or pantoprazole, within age strata. TB, tuberculosis.
(DOCX)

S3 Table Association between lansoprazole and incident TB disease, compared with omeprazole or pantoprazole allowing a 90-day treatment gap before assuming therapy has stopped. (DOCX)

S1 Protocol Protocols for research using the CPRD, Clinical Practice Research Datalink. (DOC)

## Abbreviations

BMI: body mass index
CKD: chronic kidney disease
COPD: chronic obstructive pulmonary disease
CPRD: Clinical Practice Research Datalink
DRTB: drug-resistant tuberculosis
HES: Hospital Episode Statistics
HR: hazard ratio
IBD: inflammatory bowel disease
ICD: International Classification of Diseases
IC_50_: lansoprazole concentration of 10 μM and half maximal activity
IMD: index of multiple deprivation
ISAC: Independent Scientific Advisory Committee
MI: myocardial infarction
MTB: *Mycobacterium* *tuberculosis*
NHS: National Health Service
PPI: proton pump inhibitor
pyrs: person-years
RA: rheumatoid arthritis
TB: tuberculosis
