# Supplementary material for: Lansoprazole use and tuberculosis incidence in the United Kingdom Clinical Practice Research Datalink: A population based cohort
Source: PLoS Med. 2017 Nov 21;14(11):e1002457. doi: 10.1371/journal.pmed.1002457 (PMC5697821; doi:10.1371/journal.pmed.1002457)
Supplement: S2 Fig — PPI, proton pump inhibitor; TB, tuberculosis. (DOCX) [file pmed.1002457.s003.docx]

**S2 Fig. Scatterplot of Practice Level TB Prevalence and Practice Level Lansoprazole Prescribing as a Proportion of PPI Prescribing**

**
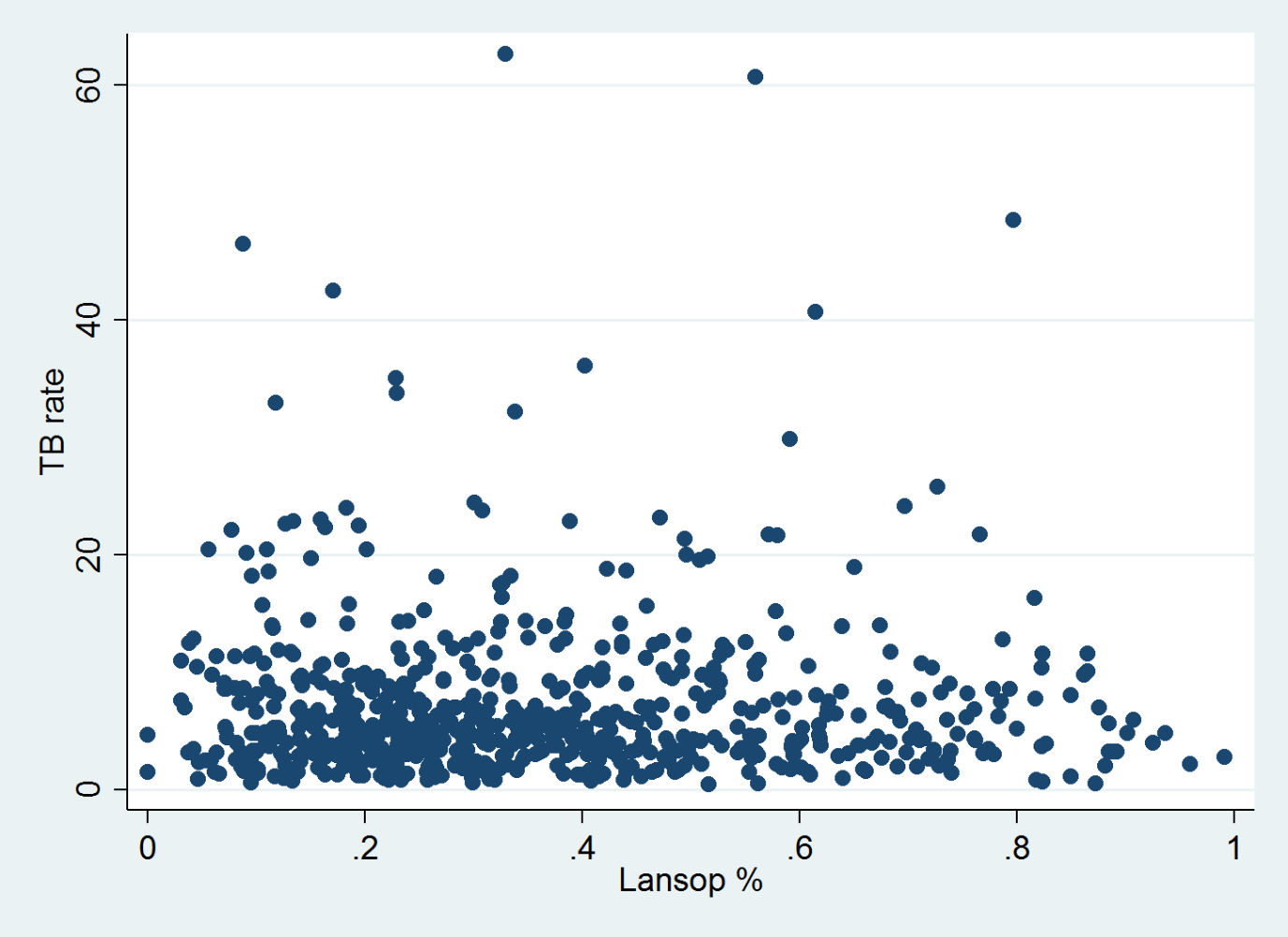
**

*TB rate – number of TB cases per 10,000 patients registered in each practice over the study period*

*Lansop % - practice level proportion of lansoprazole, omeprazole or pantoprazole prescribing accounted for by lansoprazole*
