# Supplementary material for: Lansoprazole use and tuberculosis incidence in the United Kingdom Clinical Practice Research Datalink: A population based cohort
Source: PLoS Med. 2017 Nov 21;14(11):e1002457. doi: 10.1371/journal.pmed.1002457 (PMC5697821; doi:10.1371/journal.pmed.1002457)
Supplement: S1 Text — TB, tuberculosis. (DOCX) [file pmed.1002457.s004.docx]

**S1 Text: Read Codes indicating TB disease**

| medcode | readterm |
| --- | --- |
| 635 | Pulmonary tuberculosis |
| 1840 | Tuberculosis |
| 2193 | Tuberculosis of intestines, peritoneum and mesenteric glands |
| 2208 | Tuberculosis of hip |
| 3273 | Tuberculosis of vertebral column - Pott's |
| 3303 | Tuberculosis of kidney |
| 3596 | Tuberculosis of bones and joints |
| 3830 | Tuberculosis of bladder |
| 4256 | Tuberculosis of peripheral lymph nodes |
| 4621 | Tuberculosis lichenoides |
| 4907 | Tuberculosis limb bones - Tuberculous dactylitis |
| 5145 | Tuberculosis of hilar lymph nodes |
| 6553 | Tuberculosis of spine |
| 12338 | Tuberculosis of other bones |
| 15158 | Tuberculosis NOS |
| 16331 | Tuberculosis of lung with cavitation |
| 16367 | Tuberculosis - lupus vulgaris |
| 16414 | Miliary tuberculosis |
| 16582 | TB - tuberculosis notification |
| 16996 | TB - acute pericarditis |
| 18950 | Other specified pulmonary tuberculosis |
| 23451 | Tuberculosis of knee |
| 23940 | Renal tuberculosis |
| 24372 | Tuberculosis of limb bones |
| 24413 | TB lung confirm sputum microscopy with or without culture |
| 24517 | Primary respiratory TB confirm bact and histologically |
| 26344 | Tuberculosis of peripheral lymph nodes NOS |
| 27399 | Tuberculosis of epididymis |
| 29482 | Tuberculosis of mesenteric lymph glands |
| 30687 | Tuberculosis of skin and subcutaneous tissue NOS |
| 30945 | Tuberculosis of testis |
| 31349 | Fallopian tube tuberculosis |
| 31436 | Tuberculosis of small intestine |
| 31445 | Tuberculosis verrucosa cutis |
| 31670 | Resp TB bacteriologically and histologically confirmed |
| 31844 | Acute miliary tuberculosis of a single specified site |
| 32180 | Notification of tuberculosis |
| 32459 | Other specified miliary tuberculosis |
| 33372 | Other gastrointestinal tract tuberculosis NOS |
| 34430 | Other specified tuberculosis |
| 34657 | Tuberculosis of other urinary organs |
| 35760 | Tuberculosis of adrenal glands - Addison's disease |
| 37422 | Tuberculosis of genitourinary system |
| 37598 | Tuberculosis of mediastinum |
| 37834 | Tuberculosis of pleura |
| 37886 | Tuberculosis of other specified bones |
| 38110 | Pulmonary tuberculosis NOS |
| 39279 | Tuberculosis of large intestine |
| 40231 | Tuberculosis pericardium |
| 40605 | Prim respiratory TB without mention of bact or hist confirm |
| 41208 | Tuberculosis of meninges and central nervous system |
| 41383 | Other specified tuberculosis of central nervous system |
| 42201 | Tuberculosis of other specified joint |
| 42479 | Acute miliary tuberculosis of multiple sites |
| 42630 | Other primary progressive tuberculosis |
| 43271 | Sputum: tubercle on Z-N stain |
| 43370 | Tuberculosis of spine (Pott's) |
| 43976 | Tuberculosis of eye |
| 44039 | Tuberculosis of larynx, trachea & bronchus conf bact/hist'y |
| 44128 | Tuberculosis of bones or joints NOS |
| 44129 | Tuberculosis of mediastinal lymph nodes |
| 44573 | Tuberculosis with erythema nodosum hypersensitivity reaction |
| 44655 | TB intrathoracic lymph nodes confirm bact histologically |
| 45861 | Tuberculosis of nasal sinus |
| 45932 | Tuberculosis of other organs NOS |
| 46019 | Tuberculosis of stomach |
| 46147 | Tuberculosis of liver |
| 46272 | Tuberculous pleurisy in primary progressive tuberculosis |
| 46383 | Tuberculosis of other specified organs NOS |
| 46727 | Tuberculosis of other specified organs |
| 46802 | Tuberculosis of spinal meninges |
| 46926 | Tuberculosis of intrathoracic lymph nodes NOS |
| 47336 | Lung tuberculosis |
| 47430 | Tuberculosis - scrofuloderma |
| 47881 | Tuberculosis of skin and subcutaneous tissue |
| 48580 | Nodular lung tuberculosis |
| 49433 | Tuberculosis of retroperitoneal lymph nodes |
| 49481 | Tuberculosis seminal vesicle |
| 49503 | Tuberculosis of tracheobronchial lymph nodes |
| 50147 | Other specified respiratory tuberculosis NOS |
| 50261 | Genitourinary tuberculosis NOS |
| 50489 | Tuberculosis of urinary tract |
| 50869 | Tuberculosis myocardium |
| 50902 | Other specified respiratory tuberculosis |
| 53331 | Miliary tuberculosis NOS |
| 53473 | Isolated tracheal or bronchial tuberculosis NOS |
| 53548 | Tuberculosis of ureter |
| 53701 | Infiltrative lung tuberculosis |
| 53864 | Tuberculosis of the bones of the shoulder region |
| 54570 | Other gastrointestinal tract tuberculosis |
| 54579 | Tuberculosis of gastrointestinal tract NOS |
| 54840 | Tuberculosis of cerebral meninges |
| 55298 | [X]Resp TB unspcf,w'out mention/bacterial or histol confrmtn |
| 55835 | Tuberculosis of other organs |
| 56670 | Tuberculosis oesophagus |
| 56771 | Cystitis in tuberculosis |
| 56833 | Erythema nodosum with tuberculosis NOS |
| 57587 | Tuberculosis of the lower leg bone |
| 58140 | Maternal tuberculosis,unspec whether in pregnancy/puerperium |
| 58588 | Tuberculosis of lung, confirmed by unspecified means |
| 58673 | Tuberculosis of ear |
| 58827 | Tuberculosis of intrathoracic lymph nodes |
| 59087 | Tuberculosis of rectum |
| 59916 | Tuberculosis of the pelvic and thigh bones |
| 60040 | Tuberculosis of prostate |
| 62033 | Tuberculosis papulonecrotica |
| 62468 | Tuberculosis of bronchus |
| 62530 | Tuberculosis of lung, confirmed histologically |
| 62963 | Tuberculosis of the upper arm bone |
| 63351 | Tuberculosis cutis |
| 63959 | Other respiratory tuberculosis |
| 65994 | Tuberculosis of lumbar spine |
| 66584 | Tuberculosis of other female genital organs |
| 66976 | Tuberculosis spleen |
| 67292 | Tuberculosis of kidney NOS |
| 67337 | Tuberculosis of thoracic spine |
| 67601 | Tuberculosis of the bones of the ankle and foot |
| 67637 | Tuberculosis - lupus exedens |
| 68154 | Tuberculosis of the forearm bone |
| 68821 | Tuberculosis of other male genital organs |
| 68973 | Tuberculosis of other female genital organs NOS |
| 69154 | Tuberculosis of other male genital organs NOS |
| 69260 | Isolated tracheal or bronchial tuberculosis |
| 69471 | Resp TB unspcf,w'out mention/bacterial or histol confrmtn |
| 70140 | Tuberculosis of central nervous system NOS |
| 70293 | Tuberculosis of cervical spine |
| 70491 | Tuberculosis of eye NOS |
| 70862 | Tuberculosis of bone NOS |
| 71138 | Tuberculosis of the bones of other sites |
| 72008 | Acute miliary tuberculosis |
| 72402 | Tuberculosis of nasopharynx |
| 72680 | [X]Tuberculosis of other specified organs |
| 73149 | [X]Tuberculosis |
| 73590 | Tuberculosis of thyroid gland |
| 91666 | Streptomycin resistant tuberculosis |
| 93015 | Isolated tracheal tuberculosis |
| 93071 | Tuberculosis of lung, confirmed by culture only |
| 93948 | Isolated bronchial tuberculosis |
| 94249 | Keratitis due to tuberculosis |
| 95332 | Tuberculosis of other limb bones |
| 96668 | Tuberculosis endocardium |
| 97325 | Tuberculosis of sacrum/coccyx |
| 97525 | Ciprofloxacin resistant tuberculosis |
| 97658 | Tuberculosis of nasal septum |
| 97922 | [X]Miliary tuberculosis, unspecified |
| 99188 | Maternal tuberculosis in pregnancy/childbirth/puerperium |
| 99305 | Tuberculosis of bone, site unspecified |
| 99593 | Tuberculosis of the bones of multiple sites |
| 99783 | Tuberculosis of the bones of the hand |
| 99914 | Tuberculosis of unspecified limb bone |
| 99925 | Encephalitis due to tuberculosis |
