# Supplementary material for: Lansoprazole use and tuberculosis incidence in the United Kingdom Clinical Practice Research Datalink: A population based cohort
Source: PLoS Med. 2017 Nov 21;14(11):e1002457. doi: 10.1371/journal.pmed.1002457 (PMC5697821; doi:10.1371/journal.pmed.1002457)
Supplement: S1 Table — TB, tuberculosis. (DOCX) [file pmed.1002457.s005.docx]

**S1 Table: Association between lansoprazole and incident TB disease, compared with omeprazole or pantoprazole; sensitivity analyses adjusting for variables not recorded for all patients**

| ***Sensitivity Analysis with adjustment for additional variables not recorded in all patients (complete case analysis)***  ***Using primary TB case definition (CPRD date -12 months)*** | | |
| --- | --- | --- |
| *Additional Variable*  Exposure group | **Complete case analysis adjusted for all variables in primary analysis** | **Complete case analysis adjusted for all variables in primary analysis plus additional variable** |
| *Smoking*  Omep/pantoprazole  Lansoprazole | Referent  0.68 (0.52-0.89) | Referent  0.66 (0.50-0.86) |
| *Alcohol*  Omep/pantoprazole  Lansoprazole | Referent  0.69 (0.52-0.90) | Referent  0.68 (0.52-0.90) |
| *BMI*  Omep/pantoprazole  Lansoprazole | Referent  0.70 (0.53-0.92) | Referent  0.70 (0.53-0.92) |
| *Ethnicity*  Omep/pantoprazole  Lansoprazole | Referent  0.74 (0.55-1.00) | Referent  0.74 (0.55-1.00) |
| *CKD (in people with diabetes)*  Omep/pantoprazole  Lansoprazole | Referent  0.35 (0.14-0.87) | Referent  0.35 (0.14-0.88) |

*Adjusted for all variables listed in Table 2, plus the additional variable
