# Supplementary material for: Lansoprazole use and tuberculosis incidence in the United Kingdom Clinical Practice Research Datalink: A population based cohort
Source: PLoS Med. 2017 Nov 21;14(11):e1002457. doi: 10.1371/journal.pmed.1002457 (PMC5697821; doi:10.1371/journal.pmed.1002457)
Supplement: S2 Table — TB, tuberculosis. (DOCX) [file pmed.1002457.s006.docx]

**S2 Table: Association between lansoprazole and incident TB disease, compared with omeprazole or pantoprazole, within age strata**

| *Primary analysis population and outcome definition* | |
| --- | --- |
| Age group | Adjusted* HR (lansop vs others)  (p for interaction = 0.90) |
| <=30 | 0.29 (0.03-2.40) |
| 30-40 | 0.52 (0.19-1.42) |
| 40-55 | 0.91 (0.55-1.49) |
| 55-65 | 0.71 (0.42-1.18) |
| 65-75 | 0.61 (0.36-1.01) |
| >75 | 0.58 (0.30-1.14) |

**Adjusted for all variables listed in Table 2
