# Supplementary material for: Lansoprazole use and tuberculosis incidence in the United Kingdom Clinical Practice Research Datalink: A population based cohort
Source: PLoS Med. 2017 Nov 21;14(11):e1002457. doi: 10.1371/journal.pmed.1002457 (PMC5697821; doi:10.1371/journal.pmed.1002457)
Supplement: S3 Table — (DOCX) [file pmed.1002457.s007.docx]

**S3 Table: Association between lansoprazole and incident TB disease, compared with omeprazole or pantoprazole allowing a 90 day treatment gap before assuming therapy has stopped**

| **Outcome**  **Exposure Group** | **Pyrs at risk**  **(x100,000)** | **TB cases (n)** | **Rate of TB**  **Per 100,000 pyrs** | **Crude Hazard ratio (95% CI):** | **Adjusted* HR (95% CI):** |
| --- | --- | --- | --- | --- | --- |
| ***Primary Analysis: TB date = CPRD date -12 months*** | | | | | |
| *All PPI exposure included*  Omep/pantop exposed  Lansop exposed | 13.5  9.1 | 211  95 | 15.6 (13.6-17.8)  10.3 (8.5-11.2) | Referent  0.66 (0.52-0.84) | Referent  0.69 (0.53-0.89) |
