# Supplementary material for: Lansoprazole use and tuberculosis incidence in the United Kingdom Clinical Practice Research Datalink: A population based cohort
Source: PLoS Med. 2017 Nov 21;14(11):e1002457. doi: 10.1371/journal.pmed.1002457 (PMC5697821; doi:10.1371/journal.pmed.1002457)
Supplement: S1 Protocol — (DOC) [file pmed.1002457.s008.doc]

# ISAC APPLICATION FORM

# PROTOCOLS FOR RESEARCH USING THE CLINICAL PRACTICE RESEARCH DATALINK (CPRD)

| ISAC use only:  Protocol Number  Date submitted | .............................  ............................. | **IMPORTANT**  **If you have any queries, please contact ISAC Secretariat:** ISAC[@cprd.com](mailto:Annalisa.Rubino@gprd.com) |
| --- | --- | --- |

| **Section A: The study** | | |
| --- | --- | --- |
| 1. **Study Title**   The association between lansoprazole and tuberculosis disease; a primary care based cohort study | | |
| 1. **Has any part of this research proposal or a related proposal been previously submitted to ISAC?**   Yes No  *If Yes, please provide previous protocol numbers*: | | |
| 1. **Has this protocol been peer reviewed by another Committee? (e.g. grant award or ethics committee)**   Yes No  *If Yes, please state the name of the reviewing Committee(s) and provide an outline of the review process and outcome:* | | |
| 1. **Type of Study** (please tick all the relevant boxes which apply)   Adverse Drug Reaction/Drug Safety Drug Utilisation  Disease Epidemiology  Drug Effectiveness  Pharmacoeconomics  Methodological  Health/Public Health Services Research  Post-authorisation Safety  **Other^*^**  ^*^Please specify the type of study in the lay summary | | |
| 1. **This study is intended for** (please tick all the relevant boxes which apply)**:**   Publication in peer reviewed journals  Presentation at scientific conference  Presentation at company/institutional meetings  Regulatory purposes  Other | | |
| **Section B: The Investigators** | | |
| 1. **Chief Investigator** (full name, job title, organisation name & e-mail address for correspondence- see guidance notes for eligibility)   Ian Dougl,as  CV has been previously submitted to ISAC  **CV number:** 157_15CESL  A new CV is being submitted with this protocol  An updated CV is being submitted with this protocol | | |
| 1. **Affiliation** (full address)   London School of Hygiene & Tropical Medicine, Keppel St, London, WC1E 7HT | | |
| 1. **Corresponding Applicant**     Same as chief investigator  CV has been previously submitted to ISAC  **CV number:**  A new CV is being submitted with this protocol  An updated CV is being submitted with this protocol | | |
| 1. **List of all investigators/collaborators** (*please list the full names, affiliations and e-mail addresses* of all collaborators*, *other than the Chief Investigator*)   Other investigator: Laurie Tomlinson  CV has been previously submitted to ISAC  **CV number:**  A new CV is being submitted with this protocol  An updated CV is being submitted with this protocol  Other investigator: Tom Yates  CV has been previously submitted to ISAC  **CV number:**      269_15PS  A new CV is being submitted with this protocol  An updated CV is being submitted with this protocol  Other investigator: Krishnan Bhaskaran  CV has been previously submitted to ISAC  **CV number:** 156_15CESL  A new CV is being submitted with this protocol  An updated CV is being submitted with this protocol  Other investigator: Sinead Langan  CV has been previously submitted to ISAC  **CV number:**      268_15CEP  A new CV is being submitted with this protocol  An updated CV is being submitted with this protocol  Other investigator: Sara Thomas  CV has been previously submitted to ISAC  **CV number:**      270_CESL  A new CV is being submitted with this protocol  An updated CV is being submitted with this protocol  Other investigator: Liam Smeeth  CV has been previously submitted to ISAC  **CV number:** 045_15CEPSL  A new CV is being submitted with this protocol  An updated CV is being submitted with this protocol  [Please add more investigators as necessary]**Please note that your ISAC application form and protocol* ***must*** *be copied to all e-mail addresses listed above at the time of submission of your application to the ISAC mailbox. Failure to do so will result in delays in the processing of your application.* | | |
| 1. **Conflict of interest statement*** (please provide a draft of the conflict (or competing) of interest (COI) statement that you intend to include in any publication which might result from this work)   There are no conflicts of interest to declare  **Please refer to the International Committee of Medical Journal Editors (ICMJE) for guidance on what constitutes a COI* | | |
| 1. **Experience/expertise available** (please complete the following questions to indicate the experience/expertise available within the team of investigators/collaborators actively involved in the proposed research, including the analysis of data and interpretation of results   **Previous GPRD/CPRD Studies** **Publications using GPRD/CPRD data**  None  1-3  > 3 | | |
|  | **Yes** | **No** |
| **Is statistical expertise available within the research team?**  *If yes, please indicate the name(s) of the relevant investigator(s)*  Krishnan Bhaskaran |  |  |
| **Is experience of handling large data sets (>1 million records) available within the research team?**  *If yes, please indicate the name(s) of the relevant investigator(s)*  Ian Douglas |  |  |
| **Is experience of practising in UK primary care available within the research team?**  *If yes, please indicate the name(s) of the relevant investigator(s)*  Liam Smeeth |  |  |
| 1. **References relating to your study**   Please list up to 3 references (most relevant) relating to your proposed study:  Rybniker J, Vocat A, Sala C, Busso P, Pojer F, Benjak A, Cole ST 2015 Lansoprazole is an antituberculous prodrug targeting cytochrome bc1. Nat Commun. Jul 9;6:7659. doi: 10.1038/ncomms8659.  Zenner D, Kruijshaar ME, Andrews N, Abubakar I. 2012. Risk of tuberculosis in pregnancy: a national, primary care-based cohort and self-controlled case series study. Am J Respir Crit Care Med. 185(7):779-84. doi: 10.1164/rccm.201106-1083OC. | | |
| **Section C: Access to the data** | | |
| 1. **Financial Sponsor of study**   Pharmaceutical Industry  *Please specify:*      Academia  *Please specify:*LSHTM  Government / NHS  *Please specify:*      Charity *Please specify:*  Other  *Please specify:*      None | | |
| 1. **Type of Institution carrying out the analyses**   Pharmaceutical Industry *Please specify:*      Academia *Please specify:*LSHTM  Government Department *Please specify:*      Research Service Provider *Please specify:*  NHS *Please specify:*      Other  *Please specify:* | | |

| 1. **Data source**   The sponsor has direct access to CPRD GOLD and will extract the relevant data*    A data set will be supplied by CPRD**  CPRD has been commissioned to extract the relevant data and to perform the analyses  Other *Please specify:*    *If data sources other than CPRD GOLD are required, these will be supplied by CPRD  ** Please note that datasets provided by CPRD are limited in size. Applicants should contact CPRD ([KC@CPRD.com](mailto:KC@CPRD.com)) if a dataset of >300,000 patients is required. |
| --- |
| 1. **Primary care data** (please specify which primary care data set(s) are required)   Vision only (Default for CPRD studies)  EMIS® only*  Both Vision and EMIS®*  *Note: Vision and EMIS are different clinical systems, Vision data has traditionally been used for CPRD, EMIS is currently undergoing beta-testing.*  **Investigators requiring the use of EMIS data must discuss the study with a member of CPRD staff before submitting an ISAC application*  Please list below the name of the person/s at the CPRD with whom you have discussed your request for EMIS data: |
| **Section D: Data linkage** |
| 1. **Does this protocol also seek access to data held under the CPRD Data Linkage Scheme?**   Yes*  No  If No, please move to section E.  **Investigators requiring linked data must discuss the study with a member of CPRD staff. It is important to be aware that linked data are not available for all patients in CPRD GOLD, the coverage periods for each data source may differ and charges may be applied. Please contact the CPRD Research Team on +44 (20) 3080 6383 or email* [*kc@cprd.com*](mailto:kc@cprd.com) *to discuss your requirements before submitting your application.*  Please list below the name of the person/s at the CPRD with whom you have discussed your request:  Rachel Williams  *Please note that as part of the ISAC review of linkages, the protocol may be shared - in confidence - with a representative of the requested linked data set(s) and summary details may be shared - in confidence - with the Confidentiality Advisory Group of the Health Research Authority.* |

| 1. **Please select the source(s) of linked data being requested:**   ONS Mortality Data  NCDR Cancer Registry Data*  Inpatient Hospital Episode Statistics  MINAP  Outpatient Hospital Episode Statistics  Mother Baby Link    Index of Multiple Deprivation  Townsend Score  Other** *Please specify:*  **Please note that applicants seeking access to cancer registry data must provide consent for publication of their study title and study institution on the UK Cancer Registry website. They must also complete a* ***Cancer Dataset Agreement Form*** *(available from CPRD) and provide a* ***System level Security Policy*** *for each organisation involved in the study.*  *** If “Other” is specified, please name an individual in CPRD that this linage has been discussed with.* |
| --- |
| 1. **Total number of linked datasets requested including CPRD GOLD**: 3 |
| 1. **Is linkage to a local dataset with <1 million patients being requested?**   Yes*  No  ** If yes, please provide further details:* |
| 1. **If you have requested linked data sets, please indicate whether the Chief Investigator or any of the collaborators listed in response to question 5 above, have access to any of the linked datasets in a patient identifiable form, or associated with a patient index.**   Yes*  No  ** If yes, please provide further details:* |
| 1. **Does this study involve linking to patient *identifiable* data from other sources?**   Yes  No |
| **Section E: Validation/verification** |
| 1. **Does this protocol describe a purely observational study using CPRD data (this may include the review of anonymised free text)?**   Yes*  No**  ** Yes: If you will be using data obtained from the CPRD Group, this study does not require separate ethics approval from an NHS Research Ethics Committee.*  *** No: You may need to seek separate ethics approval from an NHS Research Ethics Committee for this study. The ISAC will provide advice on whether this may be needed.* |
| 1. **Does this study require anonymised free text?**   Yes*  No  **Please note that work involving free text can only be performed on the July 2013 CPRD GOLD database build or earlier versions. CPRD can provide further advice on the use of anonymised free text.* |
| 1. **Does this protocol involve requesting any additional information from GPs?**   Yes*  No  * *Please indicate what will be required:*  Completion of questionnaires by the GP*^ψ^* Yes  No  Provision of anonymised records (e.g. hospital discharge summaries) Yes  No  Other (please describe)  *^ψ^ Any questionnaire for completion by GPs or other health care professional must be approved by ISAC before circulation for completion.* |
| 1. **Does this study require contact with patients in order for them to complete a questionnaire?**   Yes*  No  **Please note that any questionnaire for completion by patients must be approved by ISAC before circulation for completion.* |
| 1. **Does this study require contact with patients in order to collect a sample?**   Yes*  No  ** Please state what will be collected:* |
| **Section F: Signatures** |
| 1. **Signature from the Chief Investigator**   I confirm that the above information is to the best of my knowledge accurate, and I have read and understood the guidance to applicants.  Name:      Ian Douglas      Date: 8^th^ October 2015           E. signature (type name): Ian Douglas |

**Protocol Section**

The following headings **must** be used to form the basis of the protocol. Pages should be numbered. All abbreviations must be defined on first use.

1. **Lay Summary (Max. 200 words)**

A recent study demonstrated that in laboratory tests, lansoprazole appears to have potent activity against tuberculosis. Lansoprazole belongs to a class of drugs called proton pump inhibitors and is widely used to treat stomach disorders such as heartburn. It hasn’t previously been thought to have any antibiotic properties and is not used to treat infections. Other members of the proton pump inhibitor drug class were not found to have similar properties against tuberculosis. We propose a cohort study, comparing the risk of tuberculosis disease amongst people taking lansoprazole with people taking other proton pump inhibitors. If the results of the laboratory experiments have a useful clinical application, we would expect people taking lansoprazole to have a lower rate of TB. Our findings will be the first clinical evidence of the effect of lansoprazole on active tuberculosis and will help determine whether lansoprazole may be worth investigating further for this indication.

1. **Technical Summary (Max. 200 words)**

Our overall aim is to assess the effect of lansoprazole on the risk of tuberculosis disease. In order to achieve this we will estimate the relative risk of tuberculosis comparing people prescribed lansoprazole with people prescribed omeprazole or pantoprazole. We will construct a cohort of new users of lansoprazole and new users of omeprazole or pantoprazole and use Cox regression to estimate the hazard ratio for tuberculosis disease.

1. **Objectives, Specific Aims and Rationale**
2. *Aim*

To assess the effect of lansoprazole on the risk of tuberculosis (TB) disease.

1. *Objectives*

To estimate the relative risk of TB comparing people prescribed lansoprazole with people prescribed omeprazole or pantoprazole.

1. *Rationale*

By demonstrating whether the rate of TB disease is lower in people taking lansoprazole compared with omeprazole or pantoprazole we will be able to see whether this drug may be worth investigating further for clinical benefit against TB.

1. **Background**

Rybniker et al^1^ recently used an intracellular assay procedure to screen for drugs with antituberculous potential. They found that lansoprazole was highly active against TB in cell culture, when transformed intracellularly from the prodrug lansoprazole to lansoprazole sufoxide. Strong activity was also found against drug-resistant TB clinical isolates suggesting further clinical investigation is warranted. Of note, other PPIs (omeprazole and pantoprazole) were not found to have anti-tuberculous activity. To date there have been no clinical studies assessing whether lansoprazole as used in routine clinical care has such anti-tuberculous properties. Given the widespread use of PPIs over many years, we believe the CPRD provides an ideal opportunity to investigate whether lansoprazole may be associated with a lower risk of TB disease by making a direct comparison between lansoprazole users and users of omeprazole or pantoprazole.

1. **Study Type**

This is a hypothesis testing study

1. **Study Design**

This will be a cohort study comparing the rate of TB disease in people treated with lansoprazole, compared with people treated with omeprazole or pantoprazole. This design will minimise confounding because both lansoprazole exposed and unexposed patients will be receiving a PPI and will therefore have similar underlying health problems which may be associated with the risk of TB disease.

1. **Sample Size**

A feasibility count from Jan 2015 CPRD GOLD indicates the following:

- Lansoprazole new users n=744,144
- Omeprazole and pantoprazole new users n=1,182,888
- Lansoprazole new users with subsequent TB n=1,227

At this stage we cannot be sure how many incident TB cases will occur during PPI exposure, rather than after exposure has ended. Nor can we state with confidence what effect size we expect to detect. Table 1 therefore gives estimates of power under a range of assumptions, based on a conservative 1:1 comparison between lansoprazole and omeprazole or pantoprazole.

|  | *Number of exposed cases* | | | |
| --- | --- | --- | --- | --- |
| *Relative Risk* | **1,200** | **800** | **500** | **200** |
| **0.9** | 0.73 | 0.56 | 0.38 | 0.18 |
| **0.8** | >0.99 | >0.99 | 0.94 | 0.61 |
| **0.7** | >0.99 | >0.99 | >0.99 | >0.99 |
| **0.6** | >0.99 | >0.99 | >0.99 | >0.99 |
| **0.5** | >0.99 | >0.99 | >0.99 | >0.99 |

Table 1: Estimates of power to detect a range of possible relative risks, under a range of assumptions about the number of likely lansoprazole exposed TB cases

For most of the assumptions tested, we will have adequate power to detect an association with lansoprazole assuming a protective association of 0.8 or greater, which we believe would represent a clinically relevant effect size.

1. **Data Linkage Required (if applicable)**

Patient-level index of multiple deprivation data are required to assess for confounding as it is likely that risk of TB will vary by socioeconomic status. Since this is only available for patients in the linkage scheme, we propose to use practice-level index of multiple deprivation for the remainder of patients (rescaled to be on the same scale as the patient level scores, using observations where both are available). We have used this strategy successfully in previous CPRD studies.

We will also use HES (in- and out-patient data) to identify cases of TB recorded in hospital.

1. **Study Population**

The source population for this study is all patients aged 16 years or over with acceptable user status registered in the entire CPRD, with up-to-standard (uts) follow up of at least 12 months.

From the source population we will select all patients who are:

1. New users of lansoprazole; defined as people starting treatment with lansoprazole after at least 12 months uts follow up, with no previous record of receiving a PPI.
2. New users of omeprazole or pantoprazole; defined as people starting treatment with omeprazole or pantoprazole after at least 12 months uts follow up, with no previous record of receiving a PPI.

Exposure to lansoprazole, omeprazole and pantoprazole will be defined by prescription records, as conducted previously by this research group^2^.

All patients with a record of TB disease prior to their first PPI will be excluded from the study.

1. **Selection of comparison group(s) or controls**

All new users of omeprazole and pantoprazole are to be included as the comparator group, as described in Section I above. Users of a PPI are likely to be more sick than people not using PPIs, and therefore may be at an increased risk of outcomes such as acquiring an infection. Omeparazole and pantoprazole exposed people are therefore the ideal comparator group as we assume that the choice of specific PPI is less likely to be related to underlying health, and both were found to have no effect on TB in the study that motivated this work. Other PPIs were not investigated by Rybniker et al and so have not been included here. We believe it is very unlikely that PPI choice is related to the future risk of TB.

1. **Exposures, Outcomes and Covariates**

For PPI exposure, we will use recorded information on intended treatment duration and dosage instructions to estimate likely exposure duration. Where duration is missing, we will impute the population median exposure length. From past experience of PPI prescribing in CPRD, the majority of prescriptions last 30 days.

For the outcome, we will select all clinical records indicating TB as identified using codes in Appendix 1. Our primary outcome will be defined as all codes indicating TB disease. The earliest recorded date of a code indicating the outcome will be taken as the date of TB disease. A previous study using a similar approach for TB case identification in CPRD found incidence rates comparable to Public Health England figures, suggesting reliable case ascertainment^3^. However, the time between TB infection and diagnosis with TB disease is known to involve a median delay of 1.26 years, but with substantial variability^4^ and so the aetiologically relevant exposure for a protective effect to be seen is likely to be some time earlier than the recorded diagnosis. For this reason, all TB dates will be moved earlier by 12 months in the primary analysis. As sensitivity analyses, TB dates will be defined as 1) as recorded in CPRD, 2) 2 years earlier than recorded and 3) 5 years earlier than recorded. In all analyses where the TB date is moved earlier, follow up time for both TB cases and non-cases will be censored the same amount of time earlier.

CPRD linked Hospital Episodes Statistics (HES) in- and out-patient data will also be searched for codes indicating tuberculosis (A15-A19). A sensitivity analysis restricted to the CPRD HES-linked practices and time periods will be conducted, taking the earliest of CPRD or HES identified TB disease.

Covariates to be explored for their potential confounding nature will be age, sex, calendar year, smoking behaviour, body mass index, alcohol use, ethnicity, drug abuse, the use of inhaled/oral corticosteroids, travel vaccines, antimalarials, diabetes, rheumatoid arthritis, irritable bowel disease, chronic obstructive pulmonary disease, asthma, chronic kidney disease, depression, leukaemia, lymphoma, myeloma any record signifying the use of chemotherapy, a record of HIV infection and index of multiple deprivation score. Of note, ciclosporin and tacrolimus, both immunosuppressive drugs, are known to interact with omeprazole but not other PPIs. It is possible that people at higher risk of TB will therefore be given PPIs other than omeprazole and for this reason, patients with a history of use of either drug will be excluded from the study population.

1. **Data/ Statistical analysis**

Each participant’s follow up time will begin at the first prescription for lansoprazole or omeprazole/pantoprazole. All subsequent time will be classified as one of the following

- *Lansoprazole exposed* – all time covered by lansoprazole prescriptions, including a 60 day period after the estimated end of treatment date, to allow for stock piling and non-adherence
- *Omeprazole/pantoprazole exposed* – all time covered by omeprazole/pantoprazole prescriptions including a 60 day period after the estimated end of treatment date, to allow for stock piling and non-adherence. This will be the baseline against which other exposures are compared.
- *Unexposed* – all time between lansoprazole/omeprazole/pantoprazole exposure periods, or between the end of PPI exposure and the end of follow up. This time is being handled separately as it is plausible that a period of time off a PPI may be a marker of important changes in underlying health, with an associated change in the risk of TB disease

Where patients switch directly between one PPI and another, exposure will be updated to the new PPI from the date it is prescribed, assuming stock-piled drug is no longer taken.

End of follow up will be defined as the earliest of first recorded TB disease, death, transfer away, end of UTS or last data collection date.

Examples of how patient follow up time will be allocated are shown in Figure 1.

*Figure 1: Example Patient Exposure Timelines*

1. Lansoprazole exposure only

12 months follow up with no PPI

End uts

Lansoprazole prescription

EXPOSURE GROUP

Lanoprazole

1. Omeprazole or pantoprazole only

12 months follow up with no PPI

End uts

Omeprazole prescription

EXPOSURE GROUP

Omeprazole

or pantoprazole

1. Lansoprazole and omeprazole/pantoprazole

12 months follow up with no PPI

End uts

Lansoprazole prescription

Omeprazole prescription

EXPOSURE GROUP

Lansoprazole

Omeprazole

or pantoprazole

4. Lansoprazole with Breaks

12 months follow up with no PPI

End uts

Lansoprazole prescription

EXPOSURE GROUP

Lansoprazole

Unexposed

5. Lansoprazole, omeprazole/pantoprazole and breaks

12 months follow up with no lansoprazole

End uts

Pantoprazole prescription

Lansoprazole prescription

EXPOSURE GROUP

Lansoprazole

Unexposed

Omeprazole

or pantoprazole

The primary comparison of interest is the risk of TB comparing lansoprazole exposed time with omeprazole or pantoprazole exposed time. People starting treatment with omeprazole or pantoprazole but later receiving lansoprazole will transition to the lansoprazole group at that time and vice versa. Follow up for each patient will end at the earliest of; TB disease, transfer out, death or end of uts follow up.

Descriptive analyses will be performed, comparing the distribution of the following variables between patients starting lansoprazole and those starting omeprazole/pantoprazole: Age, sex, calendar year, smoking behaviour, body mass index, alcohol use, ethnicity, drug abuse, the use of corticosteroids, any record signifying any prior use of chemotherapy, travel vaccines, antimalarials, diabetes , rheumatoid arthritis, irritable bowel disease, chronic obstructive pulmonary disease, asthma, chronic kidney disease, depression, leukaemia, lymphoma, myeloma, a record of HIV infection and index of multiple deprivation score.

Cox regression will be used to estimate hazard ratios and 95% confidence intervals, comparing lansoprazole exposed time against omeprazole or pantoprazole exposed time. A crude model will be constructed with just the main exposure variable, followed by a model adjusting for potential confounders.

We will fit an interaction term to look for effect modification between lansoprazole exposure and age.

As a sensitivity analysis we will vary the time following the end of estimated treatment duration after which therapy cessation is assumed, from 60 to 90 days. A further sensitivity analysis will censor all patients after a cessation or switch of PPI therapy, (cessation defined as longer than 60 days not covered by a prescription.)

1. **Plan for addressing confounding**

Potentially confounding variables have been selected based on their possible association with the risk of TB, as listed in part K. These variables will be explored and potentially adjusted for in a multivariable Cox regression model. However, we believe substantial confounding is unlikely in this study as risk factors for TB are not expected to be associated with the choice of specific PPI, with the exception of calendar year. It is possible that rates of TB have changed over time and local prescribing guidance for preferred PPI has varied over the years mainly based on cost considerations. PPI preference could also be dictated by differential drug interactions between individual PPIs, as noted above for tacrolimus and ciclosporin and people with a history of taking either drug will be excluded.

1. **Plan for addressing missing data**

There is known missingness in the smoking and alcohol status and BMI variables. From previous experience of studies involving people taking long term medication such as PPIs it is expected that <10% of patients will have missing data. We plan to conduct a complete case analysis, which relies on the assumption that the probability of these data being missing is independent of TB risk, conditional on covariates^5^; given the small amount of missing data, any violation of the assumption is unlikely to importantly affect the results. Ethnicity is likely to have substantially more missing data. Again, we will conduct a complete case analysis with this variable included in the model, recognising the reduction in sample size will be greater and that this is a limitation of the study.

1. **Limitations of the study design, data sources and analytical methods**

It is possible that TB may go unidentified in some patients and therefore unrecorded in CPRD. Assuming this is non-differential between PPIs, this will only lead to a loss of power, rather than a biased estimate; however the power calculations we present are based on identified cases and so this is not expected to be a major limitation.

The timing of TB disease recorded in CPRD will not be the same as the date when infection actually occurred as we are detecting recorded diagnoses rather than actual time of infection. We are dealing with this by moving the date of TB disease to an earlier time and have planned sensitivity analyses varying the length of this time shift. However, PPI exposure tends to be for long periods in many patients and so it is not expected that timing inaccuracy for infection will have a substantial impact on the estimated exposure status of cases.

The recording of risk factors for TB is unlikely to be perfect. For example HIV status, drug abuse and exposure to chemotherapy may not be well recorded in primary care. However, we anticipate little confounding in this study, except by calendar time and possibly age, both of which are well recorded. Other possible sources of confounding we will be unable to address include clinical commissioning group, previous imprisonment and homelessness.

We anticipate that our estimate of PPI exposure times will be flawed to some extent due to lack of adherence. This will tend to lead to an underestimate of the effect size of any true association with lansoprazole, assuming the degree of exposure misclassification is non-differential in those people with and without subsequent TB disease.

As noted above, ethnicity will not be recorded for all patients and so the complete cases analysis including this variable may give a biased result. However, although ethnicity is a strong risk factor for TB, it is unlikely to be a strong deciding factor for specific PPI selection.

1. **Patient or user group involvement (if applicable)**

At this stage we do not plan to involve patients in the study; but depending on our findings it is possible we would seek patient engagement to help shape future research plans.

1. **Plans for disseminating and communicating study results, including the presence or absence of any restrictions on the extent and timing of publication**

All results will be presented at scientific conferences and published in a peer reviewed journal.

**Amendment**

Having conducted the study as outlined, we have found a protective association between lansoprazole and TB. As a result of this we would like to conduct a further “control” analysis to measure the association between lamsoprazole and an outcome where we do not expect to detect either a harmful or beneficial causal effect. We have selected myocardial infarction (MI) for this purpose, as we do not believe the choice of PPI will be associated with this outcome, and it is an outcome with high validity in CPRD. MI will be defined as first record of an incident MI as recorded in the CPRD. Patients with a prior record of MI will be excluded from the study population due to concerns that subsequent MI records could be either prevalent or incident.

**Amendment 2**

The control analysis for MI found no association with lansoprazole. On discussion of the results, the study team has suggested a further control analysis involving another infection outcome, herpes zoster. Herpes zoster will be defined as first record of an incident herpes zoster event as recorded in the CPRD. Patients with a prior record of herpes zoster will be excluded from the study population due to concerns that subsequent records could be either prevalent or incident. Variables considered as potential confounders will be drawn from the list of covariates considered for the analysis of TB outcomes, since this list includes all risk factors for zoster identified previously by our research group(6)

1. **References**
2. Rybniker J, Vocat A, Sala C, Busso P, Pojer F, Benjak A, Cole ST 2015 Lansoprazole is an antituberculous prodrug targeting cytochrome bc1. Nat Commun. Jul 9;6:7659. doi: 10.1038/ncomms8659.
3. Douglas IJ, Evans SJ, Hingorani AD, Grosso AM, Timmis A, Hemingway H, Smeeth L. 2012 Clopidogrel and interaction with proton pump inhibitors: comparison between cohort and within person study designs. BMJ. 345:e4388. doi: 10.1136/bmj.e4388.
4. Pealing L, Wing K, Mathur R, Prieto-Merino D, Smeeth L, Moore DA. Risk of tuberculosis in patients with diabetes: population based cohort study using the UK Clinical Practice Research Datalink. BMC Med. 2015 Jun 5;13:135. doi: 10.1186/s12916-015-0381-9.
5. Borgdorff, M. W., Sebek, M., Geskus, R. B., Kremer, K., Kalisvaart, N., & van Soolingen, D. (2011). The incubation period distribution of tuberculosis estimated with a molecular epidemiological approach. International Journal of Epidemiology, 40(4), 964–970. doi:10.1093/ije/dyr058
6. White, I.R., Carlin, J.B., Bias and efficiency of multiple imputation compared with complete-case analysis for missing covariate values, Statistics in Medicine, 2010. 29 2920--2931.
7. Forbes HJ, Bhaskaran K, Thomas SL, Smeeth L, Clayton T, Langan SM. Quantification of risk factors for herpes zoster: population based case-control study. BMJ. 2014 May 13;348:g2911. doi: 10.1136/bmj.g2911.

**Appendices**

Appendix 1

CPRD medcodes indicating tuberculosis

| medcode | readterm |
| --- | --- |
| 635 | Pulmonary tuberculosis |
| 1840 | Tuberculosis |
| 2193 | Tuberculosis of intestines, peritoneum and mesenteric glands |
| 2208 | Tuberculosis of hip |
| 3273 | Tuberculosis of vertebral column - Pott's |
| 3303 | Tuberculosis of kidney |
| 3596 | Tuberculosis of bones and joints |
| 3830 | Tuberculosis of bladder |
| 4256 | Tuberculosis of peripheral lymph nodes |
| 4621 | Tuberculosis lichenoides |
| 4907 | Tuberculosis limb bones - Tuberculous dactylitis |
| 5145 | Tuberculosis of hilar lymph nodes |
| 6553 | Tuberculosis of spine |
| 12338 | Tuberculosis of other bones |
| 15158 | Tuberculosis NOS |
| 16331 | Tuberculosis of lung with cavitation |
| 16367 | Tuberculosis - lupus vulgaris |
| 16414 | Miliary tuberculosis |
| 16582 | TB - tuberculosis notification |
| 16996 | TB - acute pericarditis |
| 18950 | Other specified pulmonary tuberculosis |
| 23451 | Tuberculosis of knee |
| 23940 | Renal tuberculosis |
| 24372 | Tuberculosis of limb bones |
| 24413 | TB lung confirm sputum microscopy with or without culture |
| 24517 | Primary respiratory TB confirm bact and histologically |
| 26344 | Tuberculosis of peripheral lymph nodes NOS |
| 27399 | Tuberculosis of epididymis |
| 29482 | Tuberculosis of mesenteric lymph glands |
| 30687 | Tuberculosis of skin and subcutaneous tissue NOS |
| 30945 | Tuberculosis of testis |
| 31349 | Fallopian tube tuberculosis |
| 31436 | Tuberculosis of small intestine |
| 31445 | Tuberculosis verrucosa cutis |
| 31670 | Resp TB bacteriologically and histologically confirmed |
| 31844 | Acute miliary tuberculosis of a single specified site |
| 32180 | Notification of tuberculosis |
| 32459 | Other specified miliary tuberculosis |
| 33372 | Other gastrointestinal tract tuberculosis NOS |
| 34430 | Other specified tuberculosis |
| 34657 | Tuberculosis of other urinary organs |
| 35760 | Tuberculosis of adrenal glands - Addison's disease |
| 37422 | Tuberculosis of genitourinary system |
| 37598 | Tuberculosis of mediastinum |
| 37834 | Tuberculosis of pleura |
| 37886 | Tuberculosis of other specified bones |
| 38110 | Pulmonary tuberculosis NOS |
| 39279 | Tuberculosis of large intestine |
| 40231 | Tuberculosis pericardium |
| 40605 | Prim respiratory TB without mention of bact or hist confirm |
| 41208 | Tuberculosis of meninges and central nervous system |
| 41383 | Other specified tuberculosis of central nervous system |
| 42201 | Tuberculosis of other specified joint |
| 42479 | Acute miliary tuberculosis of multiple sites |
| 42630 | Other primary progressive tuberculosis |
| 43271 | Sputum: tubercle on Z-N stain |
| 43370 | Tuberculosis of spine (Pott's) |
| 43976 | Tuberculosis of eye |
| 44039 | Tuberculosis of larynx, trachea & bronchus conf bact/hist'y |
| 44128 | Tuberculosis of bones or joints NOS |
| 44129 | Tuberculosis of mediastinal lymph nodes |
| 44573 | Tuberculosis with erythema nodosum hypersensitivity reaction |
| 44655 | TB intrathoracic lymph nodes confirm bact histologically |
| 45861 | Tuberculosis of nasal sinus |
| 45932 | Tuberculosis of other organs NOS |
| 46019 | Tuberculosis of stomach |
| 46147 | Tuberculosis of liver |
| 46272 | Tuberculous pleurisy in primary progressive tuberculosis |
| 46383 | Tuberculosis of other specified organs NOS |
| 46727 | Tuberculosis of other specified organs |
| 46802 | Tuberculosis of spinal meninges |
| 46926 | Tuberculosis of intrathoracic lymph nodes NOS |
| 47336 | Lung tuberculosis |
| 47430 | Tuberculosis - scrofuloderma |
| 47881 | Tuberculosis of skin and subcutaneous tissue |
| 48580 | Nodular lung tuberculosis |
| 49433 | Tuberculosis of retroperitoneal lymph nodes |
| 49481 | Tuberculosis seminal vesicle |
| 49503 | Tuberculosis of tracheobronchial lymph nodes |
| 50147 | Other specified respiratory tuberculosis NOS |
| 50261 | Genitourinary tuberculosis NOS |
| 50489 | Tuberculosis of urinary tract |
| 50869 | Tuberculosis myocardium |
| 50902 | Other specified respiratory tuberculosis |
| 53331 | Miliary tuberculosis NOS |
| 53473 | Isolated tracheal or bronchial tuberculosis NOS |
| 53548 | Tuberculosis of ureter |
| 53701 | Infiltrative lung tuberculosis |
| 53864 | Tuberculosis of the bones of the shoulder region |
| 54570 | Other gastrointestinal tract tuberculosis |
| 54579 | Tuberculosis of gastrointestinal tract NOS |
| 54840 | Tuberculosis of cerebral meninges |
| 55298 | [X]Resp TB unspcf,w'out mention/bacterial or histol confrmtn |
| 55835 | Tuberculosis of other organs |
| 56670 | Tuberculosis oesophagus |
| 56771 | Cystitis in tuberculosis |
| 56833 | Erythema nodosum with tuberculosis NOS |
| 57587 | Tuberculosis of the lower leg bone |
| 58140 | Maternal tuberculosis,unspec whether in pregnancy/puerperium |
| 58588 | Tuberculosis of lung, confirmed by unspecified means |
| 58673 | Tuberculosis of ear |
| 58827 | Tuberculosis of intrathoracic lymph nodes |
| 59087 | Tuberculosis of rectum |
| 59916 | Tuberculosis of the pelvic and thigh bones |
| 60040 | Tuberculosis of prostate |
| 62033 | Tuberculosis papulonecrotica |
| 62468 | Tuberculosis of bronchus |
| 62530 | Tuberculosis of lung, confirmed histologically |
| 62963 | Tuberculosis of the upper arm bone |
| 63351 | Tuberculosis cutis |
| 63959 | Other respiratory tuberculosis |
| 65994 | Tuberculosis of lumbar spine |
| 66584 | Tuberculosis of other female genital organs |
| 66976 | Tuberculosis spleen |
| 67292 | Tuberculosis of kidney NOS |
| 67337 | Tuberculosis of thoracic spine |
| 67601 | Tuberculosis of the bones of the ankle and foot |
| 67637 | Tuberculosis - lupus exedens |
| 68154 | Tuberculosis of the forearm bone |
| 68821 | Tuberculosis of other male genital organs |
| 68973 | Tuberculosis of other female genital organs NOS |
| 69154 | Tuberculosis of other male genital organs NOS |
| 69260 | Isolated tracheal or bronchial tuberculosis |
| 69471 | Resp TB unspcf,w'out mention/bacterial or histol confrmtn |
| 70140 | Tuberculosis of central nervous system NOS |
| 70293 | Tuberculosis of cervical spine |
| 70491 | Tuberculosis of eye NOS |
| 70862 | Tuberculosis of bone NOS |
| 71138 | Tuberculosis of the bones of other sites |
| 72008 | Acute miliary tuberculosis |
| 72402 | Tuberculosis of nasopharynx |
| 72680 | [X]Tuberculosis of other specified organs |
| 73149 | [X]Tuberculosis |
| 73590 | Tuberculosis of thyroid gland |
| 91666 | Streptomycin resistant tuberculosis |
| 93015 | Isolated tracheal tuberculosis |
| 93071 | Tuberculosis of lung, confirmed by culture only |
| 93948 | Isolated bronchial tuberculosis |
| 94249 | Keratitis due to tuberculosis |
| 95332 | Tuberculosis of other limb bones |
| 96668 | Tuberculosis endocardium |
| 97325 | Tuberculosis of sacrum/coccyx |
| 97525 | Ciprofloxacin resistant tuberculosis |
| 97658 | Tuberculosis of nasal septum |
| 97922 | [X]Miliary tuberculosis, unspecified |
| 99188 | Maternal tuberculosis in pregnancy/childbirth/puerperium |
| 99305 | Tuberculosis of bone, site unspecified |
| 99593 | Tuberculosis of the bones of multiple sites |
| 99783 | Tuberculosis of the bones of the hand |
| 99914 | Tuberculosis of unspecified limb bone |
| 99925 | Encephalitis due to tuberculosis |
