# Supplementary material for: Lansoprazole use and tuberculosis incidence in the United Kingdom Clinical Practice Research Datalink: A population based cohort
Source: PLoS Med. 2017 Nov 21;14(11):e1002457. doi: 10.1371/journal.pmed.1002457 (PMC5697821; doi:10.1371/journal.pmed.1002457)
Supplement: Supplementary file 8 [file pmed.1002457.s002.docx]

S1 Fig: Diagrammatic representation of typical patient timelines in the primary analysis

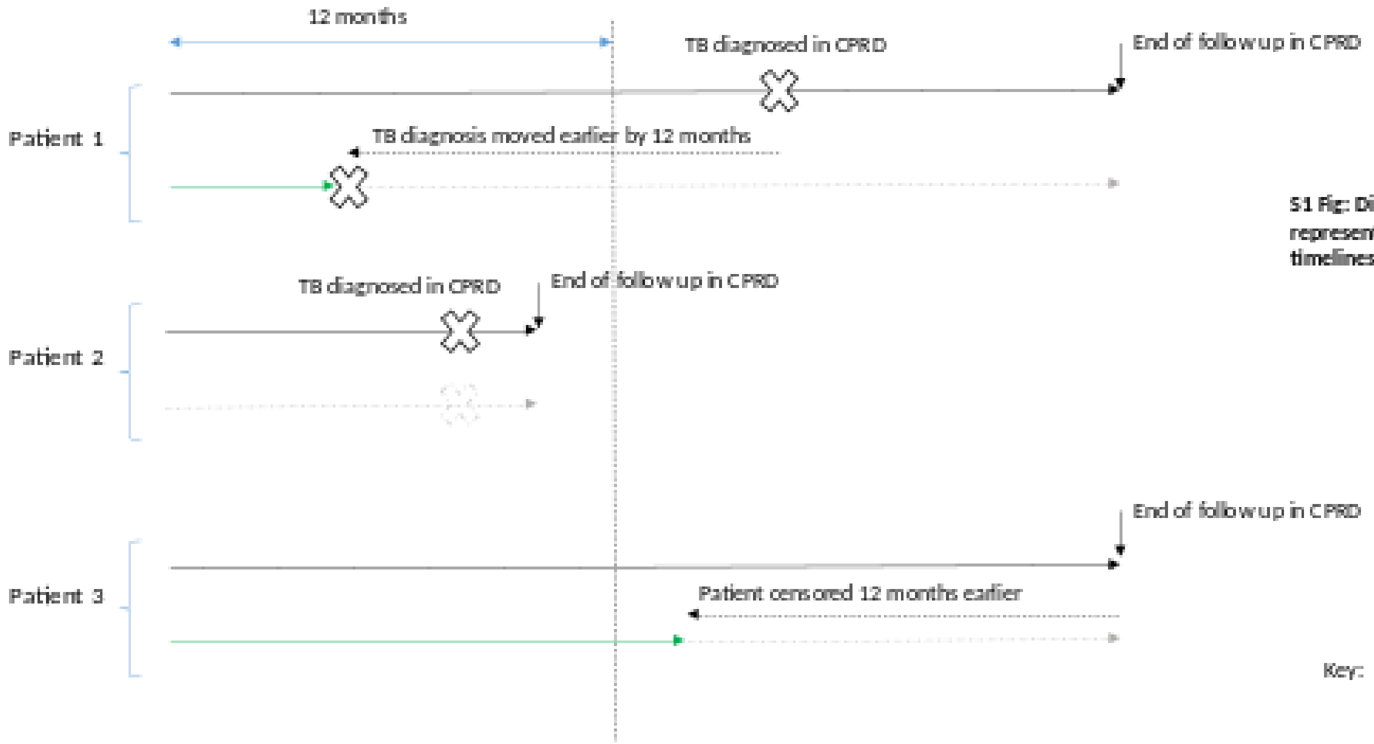

Key: TB diagnosis

Time included in CPRD

Time included in analysis

Time excluded in analysis

Patient 1 has a diagnosis of TB 16 months after starting a PPI. This diagnosis date is moved to 12 months earlier and the patient is accordingly censored after 4 months in follow up

Patient 2 has a TB diagnosis 8 months after starting a PPI. As this is <12 months after starting a PPI this patient's entire follow up is excluded from the analysis and the outcome is not counted

Patient 3 is followed for 26 months after starting a PPI, with no diagnosis of TB. Their censor date is moved 12 months earlier, as post-follow up TB diagnoses which could have been re-dated to the final 12 months follow up could not be ascertained.
